# Oral health in children and adolescents with juvenile idiopathic arthritis – a systematic review and meta-analysis

**DOI:** 10.1186/s12903-019-0965-4

**Published:** 2019-12-19

**Authors:** Marit S. Skeie, Elisabeth G. Gil, Lena Cetrelli, Annika Rosén, Johannes Fischer, Anne Nordrehaug Åstrøm, Keijo Luukko, Xieqi Shi, Astrid J. Feuerherm, Abhijit Sen, Paula Frid, Marite Rygg, Athanasia Bletsa

**Affiliations:** 1 Department of Clinical Dentistry, Pediatric Dentistry, The Faculty of Medicine, University of Bergen, Norway, Årstadveien 19, N-5009 Bergen, Norway; 2 Center for Oral Health Services and Research, Mid-Norway (TkMidt), Trondheim, Norway; 3 Department of Oral and Maxillofacial Surgery, Haukeland University Hospital, Bergen, Norway; 4 Section of Oral Diagnostics and Surgery, Department of Dental Medicine, Karolinska Institutet, Huddinge, Sweden; 5 Department of Public health and Nursing, Faculty of Medical and Health Sciences, Norwegian University of Science and Technology, Trondheim, Norway; 6 Public Dental Health Service Competence Centre of Northern Norway (TkNN), Tromso, Norway; 7 Department of Clinical Medicine, Faculty of Health Sciences, UiT, The Arctic University of Norway, Tromso, Norway; 8 Department of Otorhinolaryngology, Division of Oral and Maxillofacial Surgery, University Hospital North, Tromso, Norway; 9 Department of Clinical and Molecular Medicine, NTNU - Norwegian University of Science and Technology, Trondheim, Norway; 10 Department of Pediatrics, St. Olavs Hospital, Trondheim, Norway; 11 Oral Health Centre of Expertise in Western Norway- Hordaland, Trondheim, Norway

**Keywords:** Stomatognathic diseases, Temporomandibular joint disease, Arthritis juvenile rheumatoid, Juvenile idiopathic arthritis, Child, Adolescent

## Abstract

**Background:**

Observational studies examining the association between oral health and juvenile idiopathic arthritis (JIA) among children and adolescents have reported inconsistent findings. The aims of this systematic review and meta-analysis were to ascertain a potential difference in oral health and oral health-related quality of life (OHRQoL) among children and adolescents with JIA and healthy peers, and to assess the association of prevalence of oral diseases/conditions, temporomandibular disorders (TMD), including temporomandibular joint (TMJ) diseases, in relation to activity and severity of JIA.

**Method:**

Medline Ovid, Embase, CINAHL, SweMed+ and Cochrane Library were searched up to 25 November 2018. All articles published in English, German and Scandinavian languages focusing on children and adolescents with JIA and without JIA in relation to oral health measures, were considered. Two authors independently evaluated observational studies for inclusion. The study quality was assessed using modified Newcastle Ottawa Scale. Meta-analysis was performed for studies focusing on dental caries as an outcome.

**Results:**

Nineteen articles met the inclusion criteria, covering a range of oral diseases/conditions and OHRQoL. Eighteen studies had cross-sectional design. No mean difference of dmft/DMFT indices (decayed/missed/filled teeth) was observed between the JIA - and healthy group. None of the oral health measures including dental erosive wear, enamel defects, dental maturation and OHRQoL, indicated better oral health among children and adolescents with JIA compared to healthy group. However, periodontal conditions and TMD were more predominant among children and adolescents with JIA compared to healthy peers.

**Conclusions:**

Based on the cross-sectional studies, periodontal diseases and TMD were found to be more frequent in children and adolescents with JIA compared to healthy peers. Furthermore, more high-quality studies with large sample size are needed before we infer any concrete conclusion regarding the association between the prevalence of oral and TMJ diseases or oral conditions in relation to activity and severity of JIA.

## Background

Juvenile idiopathic arthritis (JIA) is a common chronic rheumatic condition, affecting around 1 in 1000 children under the age of 16 years [1, 2]. The incidence and prevalence of JIA varies across different studies globally, but by pooling data from several studies, it is estimated that around 60,000 children below 16 years are affected in Europe, with an estimated incidence of around 7000 new cases each year [3]. The incidence in the Nordic countries including Norway is among the highest in the world [4]. JIA comprises a group of distinct clinical entities of unknown aetiology, characterized by joint inflammation with symptoms persisting for more than six weeks and onset before 16 years of age [5]. Currently, it is classified according to the International League of Associations of Rheumatology (ILAR) as systemic arthritis, polyarthritis (Rheumatoid factor (RF) negative or positive), oligoarthritis (persistent or extended), enthesitis-related arthritis, psoriatic arthritis and undifferentiated arthritis [5].

Long-term inflammation and use of anti-inflammatory drugs, such as corticosteroids, may cause disturbances in growth and pubertal development, overall bone maturation, and eventually the development of osteopenia with low bone mineral content and low mineral density. These consequences are found to be associated with the duration of active JIA and severity [6] and are more frequent in individuals with early-onset JIA [7].

Overload of bacteria is considered as a possible trigger of rheumatic arthritis (RA) in adults [8]. This means that the oral cavity, one of the most bacteria colonised parts of the body and hosting nearly 800 species of bacteria [9], should be kept free from oral diseases. When the oral microbiota [10] shifts from balance to imbalance (dysbiosis), e.g. during rapid caries development, bacteria might pass through exposed dentine, pulp or periapical bone to the bloodstream [11]. In case of plaque accumulation at gingival margins or during ongoing gingivitis or periodontitis, bacteria might pass the blood stream through periodontal pockets, or through the oral mucosa directly if there is oral mucositis or ulcer. In patients with RA, dysbiosis has been detected in the gut and oral microbiomes (dental and saliva microbiome) and has been found to be correlated with clinical measures of RA status and to be altered compared with healthy individuals [12].

Individuals with JIA may be subjected to unfavourable underlying oral health determinants. If JIA reduces functional ability of the upper limbs, effective tooth brushing and plaque removal will be difficult. Plaque removal might also be impeded in children with JIA with restriction in mouth opening [13]. When JIA is accompanied by impaired masticatory function, consumption of softer and more sugary foods in small amounts might be more convenient [14]. Frequent and long-term intake of liquid oral medication with sugary or acidic content has previously been reported in children with JIA [15], but today sugar-free alternatives exist [16], and there is reason to believe that such intake is more rare. Knowledge of intra-oral adverse effects and frequency of side effects of modern long-term administration of anti-rheumatic drugs, is hitherto scarce.

Temporomandibular disorder (TMD) is an umbrella term including Temporomandibular joint (TMJ) involvement as well as localized pain in the masticatory muscles, decreased mouth opening and chewing ability, pain associated with mandibular movement during eating, chewing or yawning, and comorbidities such as earache and headache [7]. A high proportion of children with JIA might have involvement of the TMJ during disease course [17]. The consequences of local inflammation in the TMJs may involve local growth disturbances and as a consequence impaired mandibular growth [18]. Development of malocclusion and facial deformities such as micro- or retrognathia, are later scenarios associated with established permanent sequelae in the TMJ [7]. To identify TMJ arthritis early enough to prevent permanent growth disturbances and joint damage, it is important to recognize all clinical symptoms associated with JIA involvement. A challenge is that TMJ arthritis might evolve without or with TMD symptoms, especially in the youngest children who are unable to communicate and localize their pain adequately [7]. Thus, early detection of TMD by imaging signs of inflammation in the joints is essential.

In 2016, the key part of Vision 2020 [19] was approved, including an upgraded definition of oral health which is estimated to be multifaceted and to include different attributes of oral health. The new definition not only includes disease and condition status, but also underlying determinants, moderating factors, overall health and well-being. Thus, the ability to speak, smile, smell, taste, touch, chew, swallow and express emotions, functioning without feeling pain or discomfort, are integrated components in oral health. Children and adolescents with TMJ arthritis, may experience reductions in one, some or all these abilities, resulting in both reduced quality of life (QoL) [20] and reduced oral health-related quality of life (OHRQoL) [21]. For the group of children and adolescents with JIA, documentation of reduced OHRQoL due to oral diseases restricted to the oral cavity, e.g. dental caries, dental erosion, and not including jaw symptoms, is sparse.

Whether children and adolescents with JIA have a heavier burden of oral conditions and as a consequence, experience reduced OHRQoL, is not clearly established. For this reason, the aims of this systematic review were to gain reliable information on the following research questions;

1. Is oral health and oral health-related quality of life poorer among children and adolescents with JIA than among their healthy peers?
2. Does the activity and severity of JIA have any impact on the prevalence of oral and TMJ diseases or oral conditions?

## Methods

A systematic electronic literature search in the five main databases, Medline Ovid, Embase, CINAHL, SweMed+ and Cochrane Library, took place during the period 24.11.2017–01.12.2017. The search was later updated 25 Nov 2018. The search consisted of a combination of free text words and subject headings (i.e. MeSH, Emtree). In addition, manual searches in the reference lists of included articles were conducted. The details of search terms used for the different databases are presented in Additional file Table 1: S1.

**Table 1:** Characteristics of the studies (*n* = 19), restricted to variables selected to be included in this review

# Study	Country	Age (years)	Sample size	Study design	Oral health parameters
Ahmed N et al. 2004 [22]	UK	4–16	55 JIA (34 girls) 55 No JIA	Cross-sectional	Dental caries Oral hygiene Periodontal conditions Enamel defect TMJ dysfunction
Feres de Melo AR et al. 2014 [23]	Brazil	6–12	36 JIA (20 girls) 36 No JIA (19 girls)	Cross sectional	Dental caries Oral hygiene Periodontal conditions
Lehtinen A et al. 2000 [24]	Finland	6–14	168 JIA (95 girls) 168 No JIA (102 girls)	Cross-sectional	Tooth calcification (dental maturation)
Leksell E et al. 2008 [25]	Sweden	10–19	41 JIA (29 girls) 41 No JIA(25 girls)	Cross-sectional	Oral hygiene Periodontal conditions Oral ulcerations
Miranda LA et al. 2003 [26]	Brazil	Adolescents	32 JIA (69% girls) 24 No JIA (50% girls)	Cross-sectional	Oral hygiene Periodontal conditions
Pugliese C et al. 2016 [27]	Brazil	Adolescents	35 JIA (all girls) 35 No JIA (all girls)	Cross-sectional	Oral hygiene Periodontal conditions
Reichert S et al. 2006 [28]	Germany	12–19	78 JIA (58% girls) 75 No JIA (45% girls)	Cross-sectional	Oral hygiene Periodontal conditions
Santos D et al. 2015 [29]	Brazil	6–14	14 JIA (77% girls) 15 No JIA (48% girls)	Cross-sectional	Caries Oral hygiene Periodontal conditions TMD OHRQoL
Savioli C et al. 2004 [30]	Brazil	4.7–20	36 JIA (26 girls) 13 No JIA (9 girls)	Cross-sectional	Caries Oral hygiene Periodontal conditions
Welbury RR et al. 2003 [31]	UK	0–17 (adults included in the sample)	149 JIA (107 girls) 149 No JIA (107 girls)	Cross-sectional	Caries Oral hygiene Periodontal conditions
Miranda LA et al. 2005 [32]	Brazil Sweden	Adolescents	38 JIA 29 No JIA	Cross-sectional	Oral hygiene Periodontal conditions
Miranda LA et al. 2006 [33]	Brazil	Adolescents	18 JIA (9 girls) 14 No JIA (5 girls)	Longitudinal (2-yr-follow-up)	Oral hygiene Periodontal conditions
Maspero C et al. 2017 [34]	Italy	10–18	40 JIA 20 No JIA	Cross-sectional	Oral hygiene Periodontal conditions
Al-Shwaikh H et al. 2016 [35]	Latvia	< 17	65 JIA (45 girls) 30 No JIA (24 girls):	Cross-sectional	TMJ destruction features
Abdul-Aziez OA et al. 2010 [36]	Egypt	7.5–17.0	20 JIA (12 girls) 10 No JIA (6 girls)	Cross-sectional	TMJ inflammation
Mohammed Y et al. 2012 [37]	Egypt	8.5–17	40 JIA (26 girls) 10 No JIA (6 girls)	Cross sectional	TMJ inflammation
Leksel E et al. 2012 [38]	Sweden	10–19	41 JIA (29 girls) 41 No JIA (29 girls)	Cross-sectional	Orofacial pain TMJ
Kobus A et al. 2017 [39]	Poland	6–18	34 JIA (21 girls) 34 No JIA (21 girls)	Cross-sectional	Dental caries Oral hygiene Periodontal conditions
Ley M et al. 2009 [40]	Germany	7–17	64 JIA (48 girls) No JIA: Norms (healthy) at same age	Cross-sectional	Tooth calcification (dental maturation)

### Inclusion and exclusion criteria

This review primarily reports articles restricted to peer-reviewed journal articles published in English, German, Norwegian, Swedish or Danish during the period 1998 through 25 Nov 2018 covering children and adolescents’ age groups. Randomised controlled trials (RCTs), controlled clinical trials (CTs), cohort studies, cross-sectional studies or case-control studies were included. The exclusion criteria were systematic reviews, meta-analyses, case reports, conference publications and grey literature. Grey literature was excluded as this type can vary considerably and often be affected by low standard of quality, review and production. In addition, studies lacking comparing groups (i.e. groups without JIA) were excluded for the analyses purpose. Lastly, as another systematic review is planned, articles mainly addressing saliva variables and orthodontic considerations in children and adolescents with JIA, were excluded.

### Search strategy

PRISMA [41] was followed as a guide for reporting this systematic review and meta-analysis. The levels followed in the literature search were as follows: 1) title and authors, 2) abstracts, and 3) full text. For abstracts decided to be within the scope of interest, full-text articles were read. Two reviewers (MSS and AB) independently evaluated studies for inclusion, and studies were selected after reading abstracts, and selected full-text articles. When abstract selection was not straightforward and the reviewers were in doubt, full-text articles were re-read by both reviewers and resolved by discussion. A flow diagram is presented as Additional file 5: Figure. S1.

### Outcomes

Oral health and OHRQoL were assessed among children and adolescents with JIA and among those without JIA, and these examination data constituted the outcomes. Any outcome measures with information outside the scope of this review, but within included articles, are not mentioned. An overview of key information from the final evaluation is shown in Tables 1 and 2 in a similar way as a previous systematic review [74].

**Table 2:** Description of various ways of characterizing the studies (*n* = 19). Outcome difference is reported only between children/adolescents with JIA and those without JIA, not between subgroups of JIA patients

# Study	Matching Exclusion criteria Non-respondents: Reported (+) Not reported (−)	No of examiners Calibration	JIA assessment	Radio-graphs	Use of diagnostic tools	Outcome difference, Significantly higher: + Significantly lower: - No such difference: 0
Ahmed N et al. 2004	Gender Ethnicity Mean age: JIA: 8.9 yrs. (3.2) No JIA: 9.2 yrs. (3.2) No exclusion criteria listed Non-respondents: (−)	1 examiner Calibration: (inter- and intra-examiner results)	Not reported	No	**Caries:** dmfs/dmft, DMFS/DMFT (WHO criteria) [42] **Dental Plaque:** Plaque score (modified O’Leary index) [43] **Gingival inflammation:** Simplified Gingival Index (SGI) [43] Spontaneous gingival bleeding. **Enamel defects:** Federation Dentaire Internationale FDI notation [44] **TMJ dysfunction:** Signs and symptoms of TMJ disorders (WHO criteria) [45]	dmft/dms: 0 DMFT/DMFS: 0 Untreated caries: + Plaque: 0 Gingival inflammation: + Enamel defects: 0 TMJ dysfunction: +
Feres de Melo AR et al. 2014	Gender Mean age: JIA: 9.3 yrs. (1.9) No JIA: 9.5 yrs. (1.8) Exclusion criteria listed Non-respondents: (−)	1 examiner Calibration: (calibrated, but results not reported)	Medication	No	**Caries:** dmft/DMFT (WHO 1987) [46] **Dental Plaque:** Simplified Oral Hygiene Index (OHI-S) [47] **Gingival inflammation:** Gingival Index (GI) [48]	dmft: 0 DMFT: 0 OHI –S: + GI: 0
Lehtinen A et al. 2000	Mean age: JIA, girls: 10.4 yrs. (2.2), boys: 10.3 yrs. (2.4) No JIA, girls: 9.5 yrs. (1.6), boys: 9.8 yrs. (1.9) No exclusion criteria listed Non-respondents: (−)	1 examiner Blinded Calibration: (inter- and intra-examiner results)	Medication	OPG	**Tooth calcification (dental maturation):** Eight-stage method [49, 50]	Tooth calcification (dental maturation): +
Leksell E et al. 2008	Mean age: JIA: 13.6 yrs. (2.3) No JIA: 13.1 yrs. (1.1) Exclusion criteria listed Non-respondents: (+)	1 examiner Blinded Calibration: (inter- examination, but not reported)	Medication Disease assessment Laboratory evaluation Functional ability	BW	**Caries:** DMFS (enamel caries included) **Plaque and calculus** [51] **Bleeding on probing (BOP):** Presence or absence **Probing depth (> 2 mm) (PD):** Probe in longitudinal axis of the tooth **Clinical attachment loss (> 1 mm) (CAL):** Distance between cemento-enamel junction and the most apical portion of the probe [51] **Oral ulceration:** (discontinuation of the epithelia ≥3 mm) **Oral Questionnaire:** subjective symptoms, tooth brushing habits	DMFS: 0 Plaque: + Calculus: + BOP: + PD: + Oral ulceration: + Self-reported ulcers: + Self-reported pain or weakness in the hand: + Self-reported pain/TMD: +
Miranda LA et al. 2003	Gender Mean age: JIA: 15.9 yrs. (2.7) No JIA: 14.7 yrs. (2.3) Exclusion criteria listed Non-respondents: (−)	1 examiner Blinded Calibration: (no results)	Disease assessment Laboratory evaluation Functional ability	BW	**Dental Plaque:** Visible Plaque Index (presence or absence) **Gingival inflammation:** Gingival bleeding index (GBI) (presence or absence) **Probing depth (≥ 4 mm) (PD):** Distance between the gingival margin and the most apical portion the probe can reach **Clinical attachment level (CAL):** Distance between the cemento-enamel junction and the most apical portion the probe can reach; attachment loss (AL) when ≥2 mm	Plaque: 0 Bleeding scores: 0 PD: + AL: (≥2 mm): +
Pugliese C et al. 2016	Gender Mean age: JIA: 11.9 yrs. (2.0) No JIA: 12.5 yrs. (3.0) Exclusion criteria listed Non-respondents: (+)	Number of examiners not reported Calibration: (no results)	Medication Disease assessment Laboratory evaluation	OPG	**Caries:** DMFT **Dental Plaque:** Plaque Index (PI) [52] **Gingival inflammation:** Gingival Index (GI) Gingival bleeding index (GBI) [53] **Clinical dental attachment:** Probing Pocket Depth (PPD) **Cementoenamel Junction (CEJ):** the distance from the gingival margin to the CEJ **Clinical Attachment Level (CAL):** the sum of PPD and CEJ [54] **Radiographic evaluation of TMJ:** Orthopantomographic X-ray abnormalities (4 grades of severity) [55]	DMFT: 0 PI: 0 GI: 0 GBI: 0 PPD: 0 CEJ: + CAL: 0
Reichert S et al. 2006	Gender Ethnicity Median age: JIA: 14.4 yrs. (range 12–19) No JIA: 15.0 yrs. (range 13–19) Exclusion criteria listed Non-respondents: (−)	1 examiner Calibration: (no results)	Medication Disease assessment Laboratory evaluation	No	**Dental Plaque:** Approximal Plaque Index (API) [56] **Gingival inflammation:** Modified sulcular bleeding (GBI) [57] **Clinical attachment level:** CAL > 3.5 mm considered pathological according WHO report 1978 [58] **Assessment of CAL severity and treatment need:** Community Periodontal Index of Treatment Needs (CPITN)	API: + SBI: 0 CAL > 3.5: + CPITN: 0
Santos D et al. 2015	Mean age: JIA: 9.8 yrs. (2.86) No JIA: 10.8 yrs.(2.16) Exclusion criteria listed Non-respondents: (+)	1 examiner Calibration: (calibrated, but results not reported)	Medication Disease assessment OHRQoL (evaluated by caregivers)	No	**Caries:** dmft/DMFT **Oral hygiene:** Simplified Oral Hygiene Index (S-OHI) **Gingival inflammation:** Gingival Bleeding Index (GBI) **Signs of TMDs:** RDC/TMDS criteria **OHRQoL:** SF:13-B-PCPQ-scale [59]	dmft: - DMFT: 0 S-OHI: 0 GBI: + TMD: 0 OHRQoL: 0
Savioli C et al. 2004	No matching Median age: JIA: 10.8 yrs. (range 4.7–20) No JIA: 9.4 yrs. (range 5.4–14) No exclusion criteria listed Non-respondents: (−)	1 examiner Calibration: (calibrated, but results not reported)	Disease assessment Functional ability	No	**Caries:** DMFT, WHO criteria [45] **Oral hygiene:** Dental Plaque Index (PI) [52] **Gingival inflammation:** Gingival Bleeding Index (GBI) [60] **TMJ dysfunction:** Helkimo’s index [61]	DMFT: 0 PI: 0 GI: 0 TMJ dysfunction: +
Welbury RR et al. 2003	Gender Median age: JIA: 17.9 yrs. (range 2–50) No JIA: 10.8 yrs. Exclusion criteria listed Non-respondents: (−)	1 examiner Calibration: (inter-examiner results)	Disease assessment	No	**Caries:** dmft/DMFT (BASCD criteria) [62] **Gingival inflammation:** Gingival Index GI [48] **Oral hygiene:** The Plaque Index (PI) [63] and the Oral Cleanliness Index [64]	dmft (0–11 yrs): + DMFT: 0 D (12–17 yrs): + GI: + PI: + Oral cleanliness: -
Miranda LA et al. 2005	Mean age: JIA: 15.6 yrs. (2.7) No JIA: 14.7 yrs. (2.3) Exclusion criteria listed Non-respondents: (−)	1 examiner Blinded Calibration: (no results)	Medication Disease assessment	BW	**Dental Plaque:** Visible Plaque Index **Gingival inflammation:** Marginal Gingival Bleeding (GBI) **Probing depth (≥ 4 mm) (PD)** **Clinical Attachment Level (CAL):** Approximal attachment loss (AL) ≥2 mm	Visible plaque: 0 GBI: 0 PD > 4 mm: + AL ≥2 mm: +
Miranda LA et al. 2006	Mean age: JIA: 17.3 yrs. (2.6) No JIA: 16.6 yrs. (1.5) Exclusion criteria listed Non-respondents: (−)	2 examiners Calibration: (inter-examiner results)	Medication Disease assessment (same as above)	BW	Same variables as above	After 2-yr-follow-up: Visible plaque: 0 GB: 0 PD ≥4 mm: 0 AL ≥2 mm: 0
Maspero C et al. 2017	No matching Mean age: JIA: “Etanercept group”: 13 yrs., 10 mo JIA: “Other medication group”: 10 yrs., 11 mo No JIA: 13 yrs. 6 mo Exclusion criteria listed Non-respondents: (−)	1 examiner Calibration: (intra-examiner results)	Disease assessment	No	**Dental Plaque:** Full-Mouth Plaque Score (FMPS) **Gingival Bleeding:** Full-Mouth Bleeding Score (FMBS)	FMPS: + (highest mean in Etanercept group) GI: 0
Al Shwaikh H et al. 2016	Mean age: JIA: 14.2 yrs. (range 9–17) No JIA: 13.7 yrs. (range 10–17) Exclusion criteria not listed Non-respondents: (−)	1 examiner, supervised by radiologist. Calibration: (intra-examiner results)	Assessment of TMJ	CBCT	**TMJ destruction features** according to criteria for computerized tomography images [65]	*Condylar head:* Hypoplasia: + Subcortical sclerosis: + Subcortical cyst: + Surface flattening: + Surface erosion: + Osteophyte: + *Fossa articulare:* Surface flattening: +
Abdul-Aziez OA et al. 2010	Gender Mean age: JIA: 14.3 yrs. (2.3) No JIA: 14.5 yrs. (2.9) Exclusion criteria listed Non-respondents: (−)	Calibration: (no results)	Medication Disease assessment Laboratory evaluation Functional ability	MRI	**Clinical assessment of TMJ **[66] **Jaw mobility**: maximal interincisal mouth opening (MIO); restricted when MIO: ≤40 mm [67] **MRI evaluation of TMJ**: destruction features according MR [68]	TMJ clinical arthritis parameters: + MIO: - MR score: +
Mohammed Y et al. 2012	Gender Mean age: JIA: 14.1 yrs. (2.3) No JIA: 14.5 yrs. (2.8) Exclusion criteria listed Non-respondents: (−)	Calibration: (no results)	Disease assessment Laboratory evaluation Functional ability	MRI	**Clinical assessment of TMJ** [66] **Jaw mobility**: maximal interincisal mouth opening (MIO) **MRI evaluation of TMJ**: destruction features according MR [68]	TMJ clinical arthritis parameters: + MIO: - MR score: +
Leksel E et al. 2012	Gender Mean age: JIA: 13.6 yrs. (2.3) No JIA: 13.1 yrs. (1.1) Exclusion criteria listed Non-respondents: (+)	Examined by 1 senior orofacial pain specialist and 1 specialist in oral radiology Calibration: (no results)	Medication Disease assessment Functional ability	OPG	**Orofacial pain/TMJ pain **[69, 70] **Clinical assessment of TMJ **[71] **Radiographic evaluation of TMJ** [71]	Orofacial pain or TMJ pain: + Orofacial pain - daily life: + *Clinical findings:* Limited jaw opening: + TMJ sounds: + TMJ and muscle palpation pain: + Tender muscle sites: + Radiographic condylar changes of TMJ: +
Kobus A et al. 2017	Gender Ethnicity Mean age: JIA: 12.3 yrs. (4.6) No JIA: 12.6 yrs. (4.4) Exclusion criteria listed Non-respondents: (+)	1 examiner Calibration: (intra-examiner results)	Not reported		**Caries:** dmft/DMFT, WHO criteria [46] **Gingival inflammation**: GI index [72] **Oral hygiene (OHI-S index)** [47]	dmft: 0: DMFT: 0 GI: 0 OHI-S: 0
Ley M et al. 2009	Mean age: JIA: 12.6 yrs. (range 7.8–16.7) No JIA: norms of dental age from population studies (Germany, Holland, France-Canada) No exclusion criteria listed Non-respondents: (−)	1examiner Calibration: (intra-examiner results)	Not reported.	OPG	**Dental maturation** [73]	Dental maturation: 0

### Qualitative assessment

Central themes and topics from the PICOS (participants, interventions, comparators, and study design) approach were only to some extent extracted in Tables 1 and 2 as an intervention was not the focus. The characteristics considered important for the evaluation of reliability and validity, were study design, level of control matching, exclusion criteria, non-respondents, sample size, calibration procedures, number of examiners, documentation of JIA history (activity assessment, laboratory evaluation, medication), applied imaging type, and oral health diagnostic tools. MSS conducted the data extraction and checked by AB for accuracy. Assessment of risk of bias was performed based on an adapted version of the Newcastle – Ottawa Scale (NOS) [75], which was further modified in support of this systematic review (Additional file 2: Table S2). Scoring was performed by two authors (MSS and AB), but in case of discrepancies, a third author (AS) was consulted. The range of the scores was from 0 to 10 (low risk of bias = overall scores were 9–10, medium risk of bias = 6–8, high risk of bias = 0–5). Summarized scores of each study are presented in Additional file 3: Table S3.

### Statistical analysis

It was not possible to perform meta-analysis of oral health outcomes regarding oral hygiene (dental plaque and calculus accumulation), periodontal disease (gingivitis included), enamel defects, tooth calcification (dental maturation) disorders, TMJ arthritis, TMJ involvement, TMD, oral ulcerations, and OHRQoL. The reasons include inadequate sample size, poor study quality, use of inconsistent definitions of outcomes (e.g. periodontitis assessment) or studies that failed to report number of children and adolescents with JIA. Nevertheless, meta-analyses for dental caries was performed. Two separate meta-analyses were conducted using continuous outcomes: dmft score (decayed/missed/filled primary teeth) and DMFT score (decayed/missed/filled permanent teeth). We used random-effect model [76] to calculate pooled mean differences between dmft / DMFT scores of children and adolescents with JIA and those without JIA. The articles that did not report dmft or DMFT score or standard deviation were excluded from this meta-analysis. The heterogeneity between the studies were quantitatively assessed by the Q-test and I^2^ statistics [77]. I^2^ is the proportion of total variation explained by between-study variation. I^2^-values of 0, 25, 50% and ≥ 75% indicates no, low, moderate and high heterogeneity, respectively. Publication bias was assessed by inspection of funnel plots for asymmetry and using Egger’s test [78] and Begg-Mazumdar test [79].

A two tailed *p* < 0.05 was considered statistically significant. Statistical analyses were performed using Stata, version 15.0 software (StataCorp, Texas, USA).

## Results

Nineteen articles met the inclusion criteria, ten from Europe and nine from countries outside Europe with Brazil in the lead, see Table 1. The age range of children and adolescents with JIA was from two to four years in two studies [22, 31] and up to 20 years of age in one study [30]. Altogether, the included articles covered topics such as dental caries, oral hygiene (dental plaque and calculus accumulation), periodontal disease (gingivitis included), enamel defects, tooth calcification (dental maturation) disorders, TMJ arthritis, TMJ involvement, TMD, oral ulcerations, and OHRQoL. Beyond these topics, information about inflammatory mediator measurements in blood samples and gingival crevicular fluid, was reported.

Eighteen studies were cross-sectional in nature, only a study by Miranda et al. [32, 33] had a prospective cohort study design. At baseline, adolescents with and without JIA were examined for clinical and immunological variables of periodontal inflammation, and two years later a subgroup, eighteen adolescents with JIA and fourteen without JIA, were re-examined. Another study of Lehtinen et al. [24], distributed coded radiographs at random between different examination sessions, so the only examiner was blinded for the information of whether the radiographs belonged to participants with JIA or healthy controls.

All included studies reported age of children and adolescents with JIA and of those without JIA. However, the degree of matching varied. Although no study of true case-control design was included in the review, two studies showed controls matched for age, gender and ethnicity [22, 39]. Another characteristic of included studies was a distinct variation of sample sizes. In some studies, the sample size was too low to justify any result evidence. Beforehand sample size calculation was uncommon, as only one article [29] described this. In most studies the number of examiners was low, usually one. With some exceptions [22, 24, 31, 33], no description of calibration of examiners or reliability values were included. Bitewing radiographs were reported by only two research groups [25, 26, 32].

### Dental caries

Eight of the included articles described dental caries, but with divergent results. Both Ahmed et al. and Welbury et al. [22, 31] documented that a significantly larger proportion of children with JIA had untreated caries compared with healthy peers. Welbury et al. [31] also documented that individuals with JIA, had a higher burden of caries than individuals without JIA; among children, more primary teeth decayed, filled or extracted and among adolescents, predominantly more dental decay (D: decayed component in the DMFT). In contrast, the study of Santos et al. [29] revealed caries in primary teeth to be more frequent among healthy children than among children with JIA. Five articles [23, 25, 27, 30, 39] did not show any significant difference between the children and adolescents with and without JIA when subgroups were not included. The way of reporting caries varied from untreated caries, dmft, DMFT, D to caries prevalence of affected individuals. The use of diagnostic tools also varied. Both the World health Organization (WHO) criteria [45] and the British Association for the Study of Community Dentistry (BASCD) standardized criteria [80] were used, while some studies did not report the caries diagnostic tool used. Only one study by Leksell et al. [25], reported enamel caries.

### Quantitative synthesis

**Fig. 1:**
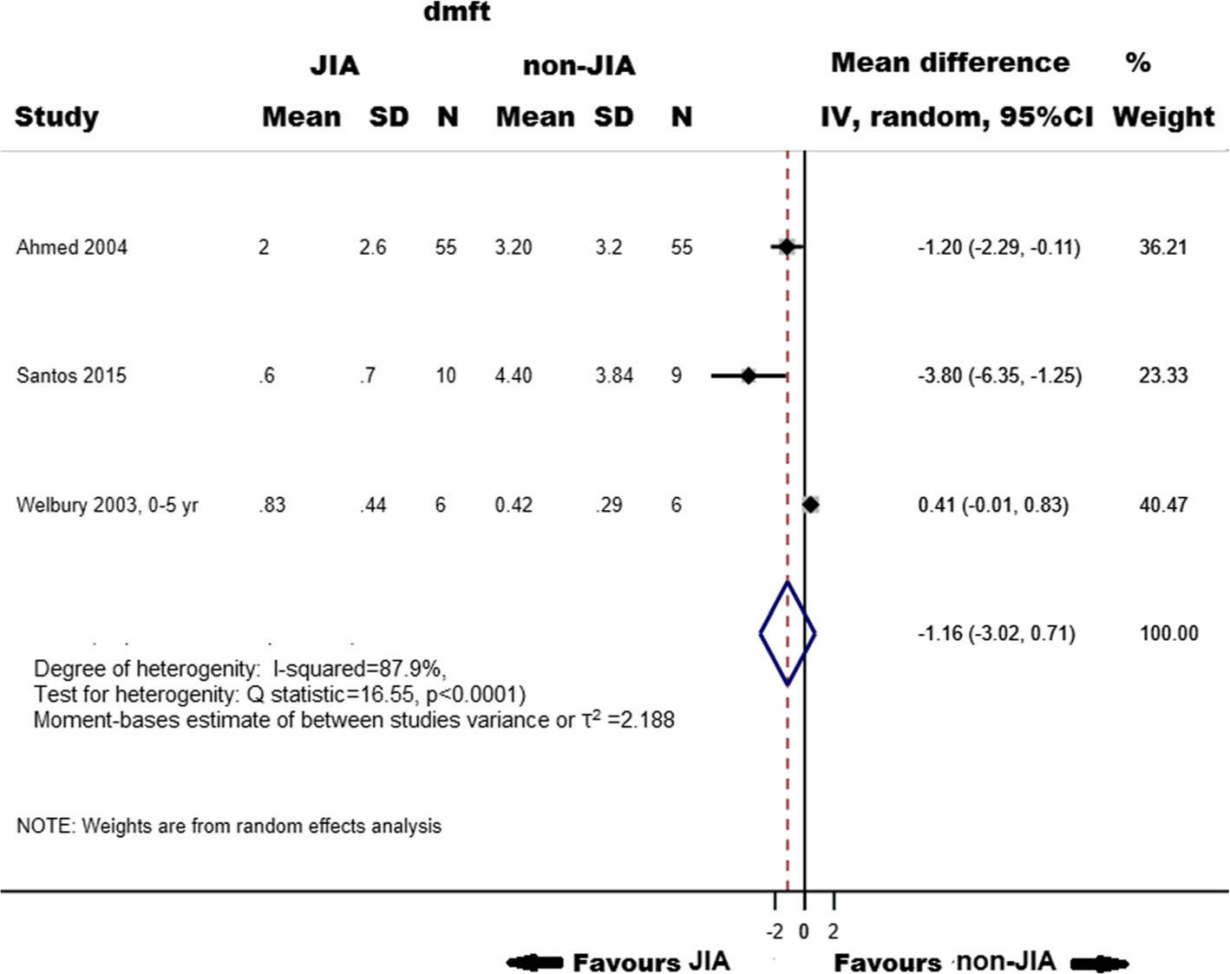
Mean differences in dmft indices in adolescents and children with Juvenile Idiopathic Arthritis (JIA) compared with those who did not experience JIA

Three cross-sectional studies (three publications) were included in the analysis to evaluate the association between caries in primary teeth and JIA (71 children with JIA and 141 total participants). We observed no difference in summary mean dmft scores between JIA and those who did not experience JIA (− 1.16, 95%CI, − 3.02-0.71, I^2^ = 87.9%, *p*_heterogenity_ = < 0.0001) (Fig. 1).

**Fig. 2:**
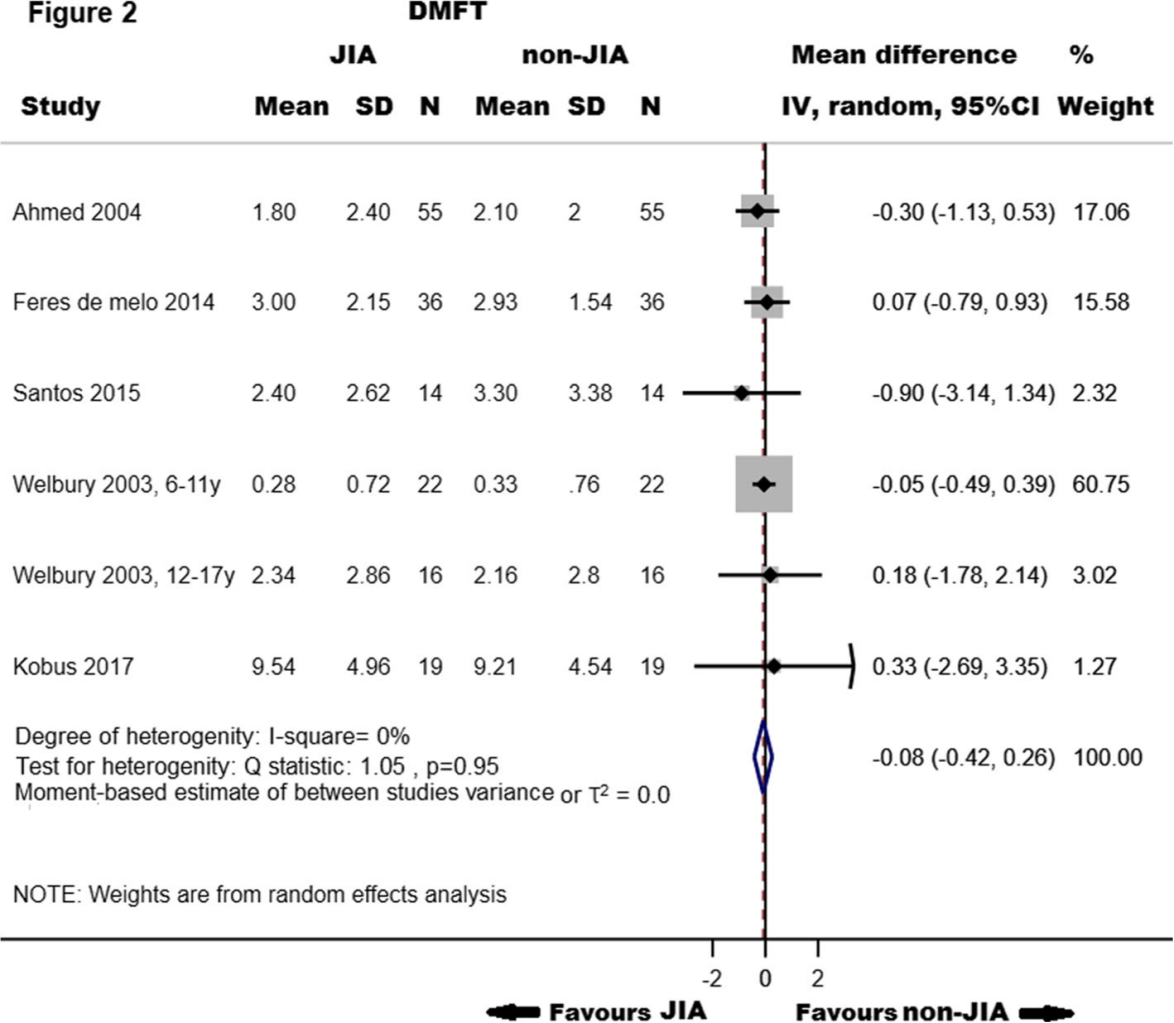
Mean differences in DMFT indices in adolescents and children with Juvenile Idiopathic Arthritis (JIA) compared with those who did not experience JIA

Six cross-sectional studies (three same publications as used above as they comprised of data from both primary- and of permanent teeth, and three other publications) were included in the analyses to evaluate the association between caries in permanent teeth and JIA (162 children and adolescents with JIA and 320 total participants). We observed no difference in summary mean DMFT score between children and adolescents with JIA and those who did not experience JIA (− 0.08, 95% CI, − 0.42 to 0.26, I^2^ statistic = 0.0%, %, *p*_heterogenity_ = 0.95) (Fig. 2).

No evidence of publication bias with Egger’s test (*P*_dmft_ = 0.27*, P*_DMFT_ = 0.78) or with Begg’s test was found (*P*_dmft_ = 0.98, *P*_DMFT_ = 0.45) (Additional file 6: Figure. S2 and Additional file 7: Figure. S3). However, because of the small number of studies and small sample size of included studies, the results from these formal tests should not be inferred with great reliability.

### Plaque, gingivitis and periodontitis

The oral health descriptors most often reported were dental plaque and signs of periodontal inflammation (gingival bleeding and bleeding on probing, probing depth ≥ 2 mm, clinical attachment loss, pocket depths etc.). There were studies focusing on oral hygiene and dental plaque [23, 25, 28, 31] showing a statistically higher Plaque Index (PI) or Simplified Oral Hygiene Index (OHI-S) in the JIA group compared with those without JIA. Other studies [22, 26, 27, 29, 30, 39] did not find this association. Additionally, Leksell et al. found calculus to be significantly more prevalent in individuals with JIA compared with those who did not experience JIA [25]. Many articles also documented poorer periodontal status among children and adolescents with JIA; more gingival inflammation and gingival bleeding [22, 29, 31], bleeding upon probing [25], deeper probing depth [25, 26] and periodontal attachment loss [26, 28]. However, not all articles documented differences in periodontal status when comparing individuals with JIA with healthy counterparts [23, 30, 39].

### Developmental enamel defects and ulcers

The only study reporting developmental enamel defect [22], found the condition more frequent among children with JIA than among healthy peers, but the sample size was too small to draw any reliable conclusion. Another study focused on oral ulceration [25] and found five out of forty-one children with JIA to be affected, but only one out of forty-one children in the group without JIA.

### Dental maturation

Two of the included studies investigated the status of dental maturation and found divergent results. By examining orthopantomograms (OPG), Lehtinen A et al. [24] in 2000 documented more advanced dental development in children with juvenile rheumatoid arthritis (JRA) compared with healthy peers, while Ley et al. [40] nine years later assessed dental maturity in children and adolescents with JIA to be within the norms of healthy peers.

### TMD

TMJ arthritis (active inflammation of the joint), TMJ involvement (osteoarthritis or growth disturbances as a result of TMJ arthritis) [81] and TMD were coherently reported more frequently among children with JIA than among healthy peers [35–38].

### OHRQoL

In the study of Leksel et al. [38], orofacial symptoms influenced more often the daily life in a group of children with JIA compared to the healthy individuals. Santos et al. [29] also compared oral health-related quality of life in children and adolescents with JIA and healthy peers. The instrument Brazilian SF:13- B-PCPQ instrument was used and consisted of thirteen items related to oral symptoms, functional limitations and well-being. In the different groups, most caregivers indicated that the oral health status of their children and adolescents had little or no effect on their well-being, and no significant differences between the groups were found.

For the present review Additional file 3: Table S3 presents the critical appraisal of the included studies while Additional file 4: Table S4 shows a completed 2009 PRISMA check list.

### Influence by JIA activity and severity

The majority of the studies contained some clinical information about the JIA status of the participants. Examples of descriptors were JIA category, disease activity, anti-rheumatic medication, JIA onset and functional impairment. Pugliese et al. [27] showed that well-established JIA disease and validated activity scores (Juvenile Arthritis Disease Activity Score (JADAS), physician global assessment of disease activity visual analogue scale (PhysglobVAS) and parent/patient global assessment of well-being VAS (PglobVAS) were positively correlated with the DMT score. Other scores; Escola Paulista de Medicina Range of Motion Scale (EPM-ROM) and Child Health Assessment Questionnaire (CHAQ) were positively correlated with Gingival Index (GI), PhysglobVAS was correlated with PI, and Pediatric Quality of Life Inventory 4.0 (PedsQL) parents was correlated with Gingival Bleeding Index (GBI). Savioli et al. [30] found that a subgroup of children with polyarticular RF negative JIA, had a statistically higher GBI and DMFT index than controls. Also a subgroup of children with three to eight affected joints in upper extremities, had significantly higher bleeding index than controls [30]. Self-reported pain or weakness in the hand when tooth brushing was documented in the study of Leksell et al. [25] as a problem among children with JIA. A significantly higher proportion of children with JIA compared with children without JIA, also answered that they did not brush their teeth when they did not feel well. Additionally, Miranda et al. reported a significantly higher mean number of joints with limitation of movements (LOM) in adolescents with two mm or more attachment loss (AL) than in adolescents without AL [26]. It is also worth mentioning the findings of Miranda et al. [32] of increased serum IL-18 and IL-1β in adolescents of JIA subgroups with AL, suggesting that AL might be associated with systemic inflammatory response. Low sample sizes, however, made it difficult to draw conclusions.

For children and adolescents with JIA, medication constitutes a substantial part of life which in turn may impact on oral health. Leksell et al. showed that children taking anti-TNFα had a higher frequency of sites with increased probing depth compared to children not taking this medicine [25]. Reichert et al. [28], comparing adolescents with JIA who took non-steroidal anti-inflammatory drugs (NSAIDs) with other JIA peers who did not take drugs, found a significant decreased mean value for modified sulcular bleeding index in the NSAID group. The frequency of cyclosporine medication, assessed by Pugliese et al. [27], was found to be higher in JIA patients with gingivitis compared with those without gingivitis. It is important to bear in mind that it is very difficult to differentiate the effect of single drugs from the effect of the disease activity with associated systemic inflammatory response in these studies. Children on anti-TNFα or cyclosporine probably have a more severe disease than children without these drugs, and differences in oral health between groups with or without a certain drug may be due to the disease severity and not the drug itself. Miranda et al. [33] in a follow-up study of adolescents with JIA documented that anti-rheumatic treatment resulting in reduction in disease activity clearly and positively influenced the periodontal status. After two years, no clinical or laboratory differences in periodontal inflammation could be documented between the adolescents with and without JIA. Pugliese et al. [27] documented that adolescents with an increased C - reactive protein (CRP) showed a higher mean clinical attachment loss (CAL) compared with those with normal CRP values.

All these reports about medication shared the previously reported problem of lacking adequate sample sizes for evidence. The comparisons were also hampered by the fact that the disease status of children and adolescents with JIA taking a certain drug were not the same as children and adolescents with JIA who did not take the drug.

## Discussion

The aim of this systematic review was to investigate the relationship between oral health measures and OHRQoL among children and adolescents with JIA compared with peers without JIA. The present systematic review and meta-analysis includes mostly studies with cross-sectional design and the overall qualitative assessment of these studies was found to be low. As a wealth of information was reported, interpretation of the data needed a clear and thorough reporting of methodology, quality and bias [82]. Tables 1 and 2 constituted the quality evidence basis when answering the research questions of the article. For dental caries, also caries meta-analysis (a quantitative method to combine data) was feasible.

Reviews published more than a decade ago have concluded that oral health in children with JIA is poor [13, 14]. However, the articles examined in these reviews were from the 1970s and the 1980s. Conclusions from this review based on more recent research on the caries situation among children and adolescents with JIA, have not been easy to draw. Due to insufficient sample size in the study of Santos et al. [29], the statistically lower dmft value among children with JIA compared with healthy peers, is not compact. The opposite conclusion drawn by Welbury et al. [31], showing a higher mean dmft among 0–11-year-olds with JIA and a higher D component among 12–17-year-olds with JIA, is probably a more reliable finding due to higher sample sizes and a calibrated examiner. Nevertheless, as the total sample included many subgroups, the exact number in the two subgroups reported was not reassuring; the youngest subgroup of both individuals with and without JIA included 46 individuals, the older subgroup 32. Additionally, bitewing radiographs were not included in the caries examination, which actually meant an underscoring of approximal caries lesions and of total caries experience [83]. However, as both cases and controls were examined without bitewing radiographs, it was not necessary to take into account any bias in the comparison.

Although the present review evaluated eight articles with caries as subject, we could not conclude that caries was more prevalent among children and adolescents with JIA than among healthy peers. The findings from this meta-analysis on dental caries suggest no significant mean difference in dmft or DMFT between JIA affected individuals and not. One of the explanations for a possible improved caries status in individuals with JIA during the later years, might be the development of a more effective overall treatment of JIA [84]. Another explanation is the increased focus on oral health in JIA, including the development of other sweeteners and sugar alternatives used in medicines e.g. in NSAID mixtures [16].

The finding that plaque, gingivitis and periodontitis were more common among children and adolescents with JIA than among those without JIA, constituted a consistent trait in the present review. The fact that so many studies drew this conclusion increased the quality of evidence supporting this result. Unfortunately, it was not possible to perform meta-analysis due to inconsistency of outcome definitions for periodontitis across the studies.

The present review lacked studies with focus on erosive wear, a condition which in later years has been reported to be as commonly distributed as caries in some groups of adolescents [85, 86]. Only one study reported on enamel defects [22], but it had very small sample size, so reliable information about the prevalence of this oral condition is still lacking. To the question of whether dental maturation was more advanced among patients with JIA than among healthy peers, there was no clear answer. The OPG radiographs in the study of Lethinen et al. [24], dating from the late 1960s to the early 1980s, were therefore too old to represent today’s patients, and the study of Ley et al. [87] instead of matched controls, compared the findings in children with JIA with normative values obtained from healthy Canadian, German and Dutch children. Conclusively, for dental erosive wear, enamel defects and dental maturation, there is no scientific evidence to answer the posed research questions.

Concerning TMJ arthritis and TMJ involvement, the present review consolidated the literature reporting these conditions to be more common in children and adolescents with JIA than in healthy counterparts [88]. However, not all the five included articles that described this topic had sufficient sample size to give reliable results [22, 35–38], but a higher frequency of surface flattening of the condylar head in children with JIA versus those without JIA, seemed to be a valid radiological feature, reported by Shwaikh et al. [35]. Furthermore, TMD and structural TMJ changes were found to be more prevalent in children with JIA than in healthy peers [38], and when comparing OHRQoL in the two groups, these were poorest among the children with JIA [38]. This was not a surprising result, taking into account that oral health definition includes all functioning without feeling pain or discomfort.

In order to answer the research question, whether OHRQoL only due to oral diseases/conditions restricted to the oral cavity is more common among those with JIA than those without, more studies related to this topic are needed. Only one study was included in the review concerning this topic: Santos et al. [29] documented that oral health status had little or no effect on well-being among both individuals with JIA and those without JIA.

To be able to respond to the second research question, whether the activity and severity of JIA had any impact on the prevalence of oral and TMJ diseases or oral conditions, larger sample sizes are needed before reliable answers can be given.

### Strengths and limitations

The strength when comparing children and adolescents with JIA with those without JIA, is that the overall oral health outcomes were taken into account and discussed elaborately. Another strength of the study was the adoption of PRISMA protocol [41] and the use of modified Newcastle-Ottawa Scale to comprehensively evaluate and assess the methodological quality of the selected studies [75]. Additionally, meta-analysis was performed for studies focusing on dental caries as an outcome. However, the present. systematic review was not without limitations. Firstly, majority of the included studies were cross-sectional in nature which is tied to high risk of bias. Secondly, due to inadequate studies and inconsistency of outcome definitions, only meta-analyses regarding dental caries, not regarding other oral diseases or conditions, could be performed. Thirdly, as grey literature was excluded in the present systematic review, the comprehensiveness of the search might have been reduced and therefore should be considered as a limitation of the review [89].

## Conclusions

Despite of limitations, periodontal diseases and TMD were found to be more frequent in children and adolescents with JIA compared with healthy peers. Regarding the association between the prevalence of oral and TMJ diseases or oral conditions in relation to activity and severity of JIA, no solid conclusion could be drawn. This systematic review and meta-analysis concluded that more high quality research with large sample size is required in this field.

## Supplementary information

**Additional file 1: Table S1.** Search history

**Additional file 2: Table S2.** Scale adapted after Newcastle-Ottawa Quality Assessment Scale for cross-sectional studies by Herzog et al. [75] and further modified in support of this systematic review

**Additional file 3:** **Table S3.** Scoring of risk of bias

**Additional file 4:** **Table S4.** 2009 PRISMA Check list

**Additional file 5:** **Figure S1.** PRISMA flow diagram of review

**Additional file 6:** **Figure S2.** Funnel plot for assessment of bias in the mean difference of dmft of primary dentition studies between children with JIA and controls (*n* = 3 studies)

**Additional file 7: Figure S3.** Funnel plot for assessment of bias in the mean difference of DMFT score of permanent dentition studies between children and adolescents with JIA and controls (*n* = 6 studies)

## Data Availability

All data generated and analysed during this study are included in this published article [and its supplementary information files].

## Abbreviations

AL: Attachment loss
BASCD: British Association for the Study of Community Dentistry
CAL: Clinical attachment loss
CHAQ: Child Health Assessment Questionnaire
CRP: C - reactive protein
CTs: Controlled clinical trials
D: Decayed component in the DMFT
DMFT: Decayed/missed/filled permanent teeth;
dmft: Decayed/missed/filled primary teeth
EPM-ROM: Escola Paulista de Medicina Range of Motion Scale
GBI: Gingival Bleeding Index
GI: Gingival Index
ILAR: International League of Associations of Rheumatology
JADAS: Juvenile Arthritis Disease Activity Score
JIA: Juvenile idiopathic arthritis
JRA: Juvenile rheumatoid arthritis
LOM: Limitation of movements
NOS: Newcastle – Ottawa Scale
NSAID: Non-steroidal anti-inflammatory drug
OHI-S: Simplified Oral Hygiene Index
OHRQoL: Oral health-related quality of life
OPG: Orthopantomograms
PedsQL: Pediatric Quality of Life Inventory 4.0
PglobVAS: Parent/patient global assessment of well-being VAS
PhysglobVAS: Physician global assessment of disease activity visual analogue scale
PI: Plaque Index
PICOS: Participants, interventions, comparators, and study design
PRISMA: Preferred reporting items for systematic reviews and meta-analyses
QoL: Quality of life
RA: Rheumatic arthritis
RCTs: Randomised controlled trials
RF: Rheumatoid factor
TMD: Temporomandibular disorder
TMJ: Temporomandibular joint
WHO: World health Organization
