# Supplementary material for: Oral health in children and adolescents with juvenile idiopathic arthritis – a systematic review and meta-analysis
Source: BMC Oral Health. 2019 Dec 19;19:285. doi: 10.1186/s12903-019-0965-4 (PMC6921440; doi:10.1186/s12903-019-0965-4)
Supplement: Supplementary file 1 — Additional file 1: Table S1. Search history [file 12903_2019_965_MOESM1_ESM.docx]

Supplementary Table 1. Search history

| **Database Medline Ovide** | |
| --- | --- |
| 1 | exp Oral health/ |
| 2 | exp stomatognathic diseases/ |
| 3 | exp tooth disease/ |
| 4 | exp mouth disease/ |
| 5 | exp stomatognathic system  abnormalities/ |
| 6 | exp stomatognathic system/ |
| 7 | exp mouth/ |
| 8 | exp Dental Care for Cronically ill/ |
| 9 | exp Periodontal index/ |
| 10 | exp Dental health surveys/ |
| 11 | exp Temporomandibular joint disorders/ |
| 12 | (temporomandibular disease or temporomandibular joint disorder or temporomandibular disorder or temporomandibular pain or oral health related quality of life or ohrqol or oral disease* or mouth or dental or periodontal or caries or gingivitis or Tooth diseases or dental enamel hypoplasia or tooth demineralization or tooth anomalies or Tooth wear or tooth erosion or Tooth abnormalitites or hypoplastic enamel or molar incisor hypomineralization).ti,ab,kw. |
| 13 | 1 or 2 or 3 or 4 or 5 or 6 or 7 or 8 or 9 or 10 or 11 or 12 |
| 14 | exp Arthritis, Juvenile/ |
| 15 | (juvenile adj3 arthritis).ti,ab,kw. |
| 16 | (JIA OR JRA).ti,ab,kw. |
| 17 | 13 OR 14 OR 15 |
| 18 | exp Child/ |
| 19 | exp Adolescent/ |
| 20 | (child OR adolescen*).ti,ab,kw. |
| 21 | 17 OR 18 OR 19 |
| 22 | 12 AND 16 AND 20 |
| 23 | limit 21 to yr=”1998-2019” |
| **Database EMBASE** | |
| 1 | (temporomandibular joint disorder or temporomandibular disease or temporomandibular disorder or temporomandibular pain or oral health related quality of life or ohrqol or oral disease* or mouth or dental or periodontal or caries or gingivitis or Tooth diseases or dental enamel hypoplasia or tooth demineralization or tooth anomalies or Tooth wear or tooth erosion or Tooth abnormalitites or hypoplastic enamel or molar incisor hypomineralization).ti,ab,kw. |
| 2 | exp tooth disease/ |
| 3 | exp mouth disease/ |
| 4 | exp stomatognathic system/ |
| 5 | exp mouth/ |
| 6 | exp dental procedure/ |
| 7 | exp periodontal index/ |
| 8 | exp dental disease assessment/ |
| 9 | 1 OR 2 OR 3 OR 4 OR 5 OR 6 OR 7 OR 8 |
| 10 | exp juvenile rheumatoid arthritis/ |
| 11 | (juvenile adj3 arthritis).ti,ab,kw. |
| 12 | (JIA OR JRA).ti,ab,kw. |
| 13 | 10 or 11 or 12 |
| 14 | exp child/ |
| 15 | exp adolescent/ |
| 16 | (child or children or adolescent*).ti,ab,kw. |
| 17 | 14 or 15 or 16 |
| 18 | 9 and 13 and 17 |
| 19 | limit 18 to yr=”1998-2019” |
| **Database Svemed+** | |
| #1 | Exp: “Temporomandibular Joint Disorders” |
| #2 | exp: “Stomatognathic Diseases” |
| #3 | exp: “Arthritis, Juvenile” |
| 4 | ((#1 OR #2) AND #3) |
| **Database Cochrane** | |
| #1 | (temporomandibular joint disorder or temporomandibular disease or temporomandibular disorder or temporomandibular pain or oral health related quality of life or ohrqol or oral disease* or mouth or dental or periodontal or caries or gingivitis or Tooth diseases or dental enamel hypoplasia or tooth demineralization or tooth anomalies or Tooth wear or tooth erosion or Tooth abnormalitites or hypoplastic enamel or molar incisor hypomineralization).ti,ab,kw. |
| #2 | MeSH descriptor: [Tooth Diseases] explode all trees |
| #3 | MeSH descriptor: [Mouth Diseases] explode all trees |
| #4 | MeSH descriptor: [Stomatognathic System] explode all trees |
| #5 | MeSH descriptor: [Temporomandibular Joint Disorders] explode all trees |
| #6 | #1 OR #2 OR #3 OR #4 OR #5 |
| #7 | MeSH descriptor: [Child] explode all trees |
| #8 | MeSH descriptor: [Adolescent] explode all trees |
| #9 | Child or children or adolescen*:ti,ab,kw (Word variations have been searched)  ., |
| #10 | #6 or #7 or 8 or #9 |
| #11 | MeSH descriptor: [Arthritis, Juvenile] explode all trees |
| #12 | JIA or JRA or (juvenile and arthritis):ti,ab,kw (Word variations have been searched) |
| #13 | #11 or #12 |
| #14 | #6 and #10 and 13 with Publication Year from 1998 to 2019. |
| **Database Cinahl** | |
| # | **Query** |
| S16 | S7 AND S11 AND S14 Date 19980101-20181231 |
| S15 | S7 AND S11 AND S14 |
| S14 | S12 AND S13 |
| S13 | child OR children OR adolescent |
| S12 | (MH “Adolescence+”) OR (MH “child+”) |
| S11 | S8 OR S9 OR S10 |
| S10 | jia OR jra |
| S9 | arthritis and juvenile |
| S8 | (MH “Arthritis, Juvenile Rheumatoid”) |
| S7 | S1 OR S2 OR S3 OR S4 OR S5 OR S6 |
| S6 | (temporomandibular joint disorder or temporomandibular disease or temporomandibular disorder or temporomandibular pain or oral health related quality of life or ohrqol or oral disease* or mouth or dental or periodontal or caries or gingivitis or Tooth diseases or dental enamel hypoplasia or tooth demineralization or tooth anomalies or Tooth wear or tooth erosion or Tooth abnormalitites or hypoplastic enamel or molar incisor hypomineralization).ti,ab,kw. |
| S5 | (MH “Dental Care+”) |
| S4 | (MH “Stomatognathic System+”) |
| S3 | (MH “Mouth Diseases+”) |
| S2 | (MH “Tooth Diseases+”) |
| S1 | (oral disease* OR mouth OR dental OR periodontal OR caries OR gingivitis OR Tooth diseases OR dental enamel hypoplasia OR tooth demineralization OR tooth anomalies OR Tooth wear OR tooth erosion OR Tooth abnormalitites OR hypoplastic enamel OR molar incisor hypomineralization).ti,ab,kw. |
