# Supplementary material for: Oral health in children and adolescents with juvenile idiopathic arthritis – a systematic review and meta-analysis
Source: BMC Oral Health. 2019 Dec 19;19:285. doi: 10.1186/s12903-019-0965-4 (PMC6921440; doi:10.1186/s12903-019-0965-4)
Supplement: Supplementary file 2 — Additional file 2: Table S2. Scale adapted after Newcastle-Ottawa Quality Assessment Scale for cross-sectional studies by Herzog et al. [75] and further modified in support of this systematic review [file 12903_2019_965_MOESM2_ESM.docx]

Supplementary Table 2. Scale adapted after Newcastle-Ottawa Quality Assessment Scale for cross-sectional studies by Herzog et al. [24] and further modified in support of this systematic review.

| **Selection: (Maximum 5 stars)** | **Scores** |
| --- | --- |
| 1. Representativeness of the target sample: |  |
| - 1. Truly representative of the average in the target population. (all subjects or random sampling) | ** |
| - 1. Partly representative of the average in the target group. | * |
| - 1. No description of included subjects. |  |
| 1. Sample size: |  |
| - 1. Justified and satisfactory (including sample size calculation). | * |
| - 1. Not justified. |  |
| - 1. No information provided |  |
| 1. Non-respondents: |  |
| - 1. Proportion of target sample recruited attains pre-specified target or basic summary of non-respondent characteristics in sampling frame recorded. | * |
| - 1. Unsatisfactory recruitment rate, no summary data on non-respondents. |  |
| - 1. No information provided |  |
| 1. Ascertainment of the group without JIA: |  |
| - 1. Health status of this group was described. | * |
| - 1. No information was provided about their health status. | * |
| **Comparability: (Maximum 2 stars)** |  |
| 1. Comparability of subjects in different outcome groups on the basis of design or analysis. Confounding factors controlled |  |
| - 1. Data/ results adjusted for relevant predictors/risk factors/confounders e.g. age, sex, etc. | ** |
| - 1. Data/results not adjusted for relevant predictors/risk factors/confounders e.g. age, sex, etc. |  |
| **Outcome: (Maximum 3 stars)** |  |
| 1. Assessment of outcome: |  |
| - 1. Independent blind assessment using objective validated diagnostic tools. | * |
| - 1. Unblinded assessment using objective validated diagnostic tools. | * |
| - 1. No description/non-standard diagnostic methods used. |  |
| 1. Calibration: |  |
| - 1. The examiners were calibrated. | * |
| - 1. No information about calibration of examiners. |  |
| 1. .Statistical test: |  |
| - 1. Statistical tests for comparisons between groups (p value). | * |
| - 1. Statistical test not appropriate, not described or incomplete. |  |
