# Supplementary material for: Oral health in children and adolescents with juvenile idiopathic arthritis – a systematic review and meta-analysis
Source: BMC Oral Health. 2019 Dec 19;19:285. doi: 10.1186/s12903-019-0965-4 (PMC6921440; doi:10.1186/s12903-019-0965-4)
Supplement: Supplementary file 3 — Additional file 3: Table S3. Scoring of risk of bias [file 12903_2019_965_MOESM3_ESM.docx]

Supplementary Table 3. Scoring of risk of bias.

|  | Selection | | | | Comparability | Outcome | | | Total | Quality category –  risk of bias |
| --- | --- | --- | --- | --- | --- | --- | --- | --- | --- | --- |
|  | Representa-  tiveness | Sample  size | Non-  respondents | Group  no JIA | Comparability | Assessment | Calibration | Statistical  tests |  | High:  *0-5*  Moderate:  *6-8*  Low:  *9-10* |
| Ahmed N et al. 2004 | 1 | 0 | 0 | 0 | 2 | 1 | 1 | 1 | 6 | Moderate |
| Feres de Melo AR et al. 2014 | 1 | 0 | 0 | 1 | 2 | 1 | 1 | 1 | 7 | Moderate |
| Lehtinen A et al. 2000 | 1 | 0 | 0 | 0 | 2 | 1 | 1 | 1 | 6 | Moderate |
| Leksell E et al. 2008 | 1 | 0 | 1 | 1 | 2 | 1 | 1 | 1 | 8 | Moderate |
| Miranda LA et al. 2003 | 1 | 0 | 0 | 1 | 2 | 1 | 0 | 1 | 6 | Moderate |
| Pugliese C et al. 2016 | 1 | 0 | 1 | 1 | 2 | 1 | 0 | 1 | 7 | Moderate |
| Reichert S et al. 2006 | 1 | 0 | 0 | 1 | 2 | 1 | 0 | 1 | 6 | Moderate |
| Santos D et al. 2015 | 1 | 0 | 1 | 1 | 2 | 1 | 1 | 1 | 8 | Moderate |
| Savioli C et al. 2004 | 1 | 0 | 0 | 1 | 0 | 1 | 1 | 1 | 5 | High |
| Welbury RR et al. 2003 | 1 | 0 | 0 | 1 | 2 | 1 | 1 | 1 | 7 | Moderate |
| Miranda LA et al. 2005 | 1 | 0 | 0 | 1 | 2 | 1 | 0 | 1 | 6 | Moderate |
| Miranda LA et al. 2006 |  |  |  |  |  |  |  |  |  |  |
| Maspero C et al. 2017 | 1 | 0 | 0 | 1 | 2 | 1 | 1 | 1 | 7 | Moderate |
| Al Shwaikh H et al. 2016 | 1 | 0 | 0 | 0 | 2 | 1 | 1 | 1 | 6 | Moderate |
| Abdul-Aziez OA et al. 2010 | 1 | 0 | 0 | 1 | 2 | 1 | 0 | 1 | 6 | Moderate |
| Mohammed Y et al. 2012 | 1 | 0 | 0 | 1 | 2 | 1 | 0 | 1 | 6 | Moderate |
| Leksel E et al. 2012 | 1 | 0 | 1 | 1 | 2 | 1 | 0 | 1 | 7 | Moderate |
| Kobus A et al. 2017 | 1 | 0 | 1 | 1 | 2 | 1 | 1 | 1 | 8 | Moderate |
| Ley M et al. 2009 | 1 | 0 | 0 | 0 | 0 | 1 | 1 | 1 | 4 | High |
