# Supplementary figures and images for: Oral health in children and adolescents with juvenile idiopathic arthritis – a systematic review and meta-analysis
Source: BMC Oral Health. 2019 Dec 19;19:285. doi: 10.1186/s12903-019-0965-4 (PMC6921440; doi:10.1186/s12903-019-0965-4)

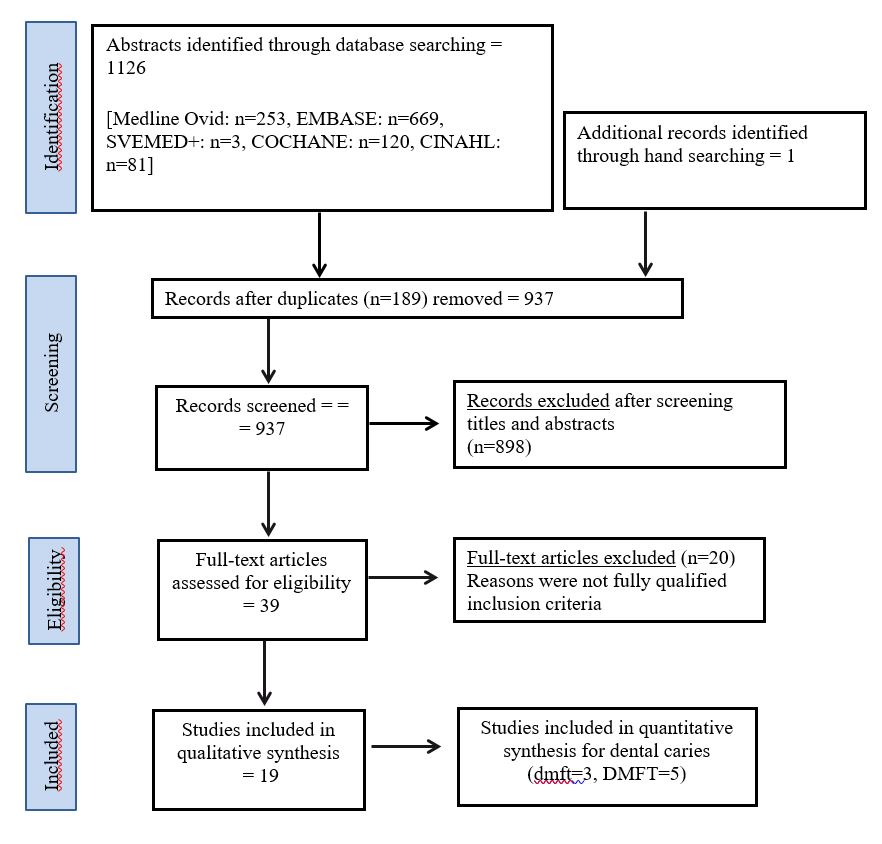

Supplement: Supplementary file 5 — Additional file 5: Figure S1. PRISMA flow diagram of review [file 12903_2019_965_MOESM5_ESM.jpg]

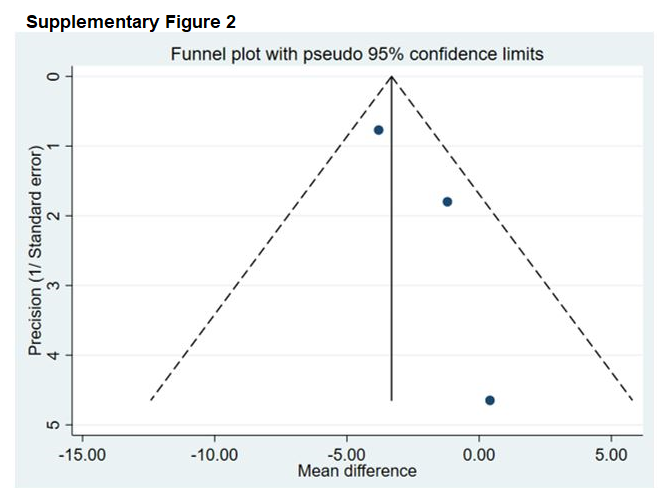

Supplement: Supplementary file 6 — Additional file 6: Figure S2. Funnel plot for assessment of bias in the mean difference of dmft of primary dentition studies between children with JIA and controls (n = 3 studies) [file 12903_2019_965_MOESM6_ESM.tif]

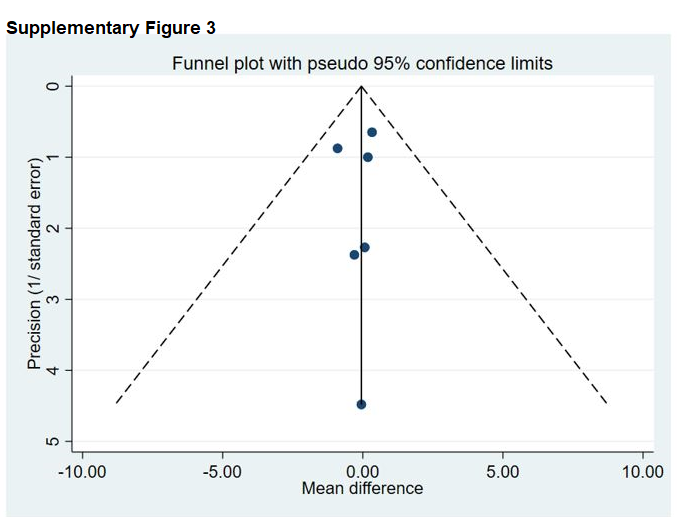

Supplement: Supplementary file 7 — Additional file 7: Figure S3. Funnel plot for assessment of bias in the mean difference of DMFT score of permanent dentition studies between children and adolescents with JIA and controls (n = 6 studies) [file 12903_2019_965_MOESM7_ESM.tif]
